# A pilot study to assess the impact of Aboriginal and Torres Strait Islander cultural humility webinars on Australian medical school students

**DOI:** 10.1186/s12909-023-04612-7

**Published:** 2023-09-03

**Authors:** R Buhagiar, A Lu, S Liu, S Sahadevan, LM Schulz, J Ghosh, A Yeoh

**Affiliations:** 1 School of Medicine, Deakin University, 75 Pigdons Road, Waurn Ponds, VIC 3216 Australia; 2 School of Medicine, The University of Melbourne, Parkville, VIC 3010 Australia; 3 School of Medicine, Monash University, Wellington Road, Clayton, VIC 3800 Australia; 4 School of Medicine, University of Queensland, St Lucia, QLD 4072 Australia; 5 School of Medicine, Western Sydney University, 255 Elizabeth Street, Sydney, NSW 2000 Australia; 6 School of Medicine, Deakin University, 75 Pigdons Road, Waurn Ponds, 3216 VIC Australia; 7 Institute of Evidence-Based Healthcare, Bond University, 14 University Drive, Robina, QLD 4226 Australia

**Keywords:** Australian Aboriginal and Torre Strait Islander Peoples, Indigenous Peoples, Curriculum, Feasibility studies, Schools, Medical

## Abstract

**Background:**

The *Aboriginal and Torres Strait Islander Health Curriculum Framework* helps higher education providers to deliver safe and well-informed cultural humility education. However, there is currently a scarcity of evidence surrounding the efficacy and impact of cultural humility education. This study will use qualitative and quantitative research methods to evaluate learning outcomes from an Indigenous health educational webinar aimed at Australian medical students.

**Methods:**

A pilot study was conducted following a group of Australian medical students who attended an educational Indigenous health (IH) culturally responsive webinar. Recruitment was conducted via the webinar hosts’ social media pages. Quantitative methods involved sending one pre- and two post-webinar questionnaires to attendees. To assess participants’ retention of information, one post-webinar survey was sent out immediately after the webinar and another three months after the webinar. These questionnaires were designed to reflect pre-determined learning objectives for the webinar. Qualitative methods involved a focus group discussion to identify common themes from participant feedback.

**Results:**

Twenty-six participants were included in the final quantitative analysis. Most of the participants were clinical students between 18 and 24 years old who did not identify as Aboriginal and/or Torres Strait Islander. There was a significant increase (*p =* 0.007) between pre-intervention (*M* = 0.35, SD = 0.26) and post-webinar knowledge for the learning outcome exploring the links between health and education (*M* = 047, SD = 0.25). No results were obtained from the three months post-intervention questionnaire. The qualitative analysis synthesized feedback from three participants and identified presenter delivery style as an important mediator of webinar effectiveness.

**Conclusions:**

There was a significant increase in knowledge and understanding for the learning outcome that explored the links between health and education. We attribute this partly to the engaging and conversational delivery style of the webinar presenters. The importance of Indigenous facilitators that encourage reflective teaching should not be understated. Our results suggest that cultural humility webinars can have a positive impact on medical students’ understanding of the Aboriginal and/or Torres Strait Islander health landscape. This pilot study warrants further research on a larger population.

**Supplementary Information:**

The online version contains supplementary material available at 10.1186/s12909-023-04612-7.

## Background

Pervasive systemic racism is a barrier to receiving health care for several Aboriginal and Torres Strait Islander peoples [1]. The importance of culturally safe practice is explicitly recognised by the *Medical Board of Australia* to prevent racism and improve health outcomes for Aboriginal and Torres Strait Islander peoples [2]. To provide culturally safe practice, medical practitioners must develop an understanding of the impact that colonisation and wider systemic racism has had on their own biases and prejudices [3]. This involves ongoing education and reflection throughout the entire career of a medical practitioner and begins in medical school [4]. The *Medical Deans of Australia and New Zealand* (MDANZ) Indigenous Health Strategy 2021–2025 outlines the education of culturally safe practitioners as a priority area for medical schools throughout Australia and New Zealand [4]. The *Aboriginal and Torres Strait Islander Health Curriculum Framework* supports this goal by providing guidance to higher education providers to develop Aboriginal and Torres Strait Islander health curricula [5].

Cultural humility education can take many forms [5]. A scoping review undertaken by Downing et al. [6] identified six models commonly used in Australian hospitals to deliver cultural humility training. The dominant model identified was ‘cultural awareness’ which focuses on Indigenous culture as opposed to context specific training for healthcare [6]. Importantly, Downing et al. [6] notes the poor quality of research evaluating the specifics of different training modalities. This conclusion is supported by two systematic reviews [7, 8]. Clifford et al. [7] aimed to describe the quality of interventions designed to improve cultural responsiveness in Australia, New Zealand, Canada, and the USA. Studies included in their review were limited by poor study design and inadequate control of confounding [6]. A larger systematic review completed by Jongen et al. [8] found inadequacies in methods of measurement when evaluating the effectiveness of cultural responsiveness interventions. They call for use of consistent evaluation approaches to better appraise intervention impact between different cultural responsiveness interventions [8].

Despite the paucity of evidence evaluating interventions related to cultural humility, there is ubiquitous recognition that evaluation of cultural awareness training programs is an important part of the process of continual improvement [8]. Identifying strategies and methods of teaching that have greater impact on participants is critical to understand how best to shift the behaviour and attitudes of participants [9]. Furthermore, short term evaluation should be supported by long term evaluation to build an understanding of sustained changes in attitudes [9]. Establishing a quality evidence base is critical to improve the delivery and impact of cultural humility training in healthcare and medical education [10]. Paul et al. [11] surveyed the impact on perceptions of cultural safety among medical students at the University of Western Australia (WA). One cohort received minimal teaching related to Aboriginal health while another cohort received extended, self-reflective and small group learning [11]. The latter group indicated a higher level of preparedness to work with Aboriginal people in clinical practice [11]. Paul et al. [11] notes that findings from this research have driven improvements in the curriculum at UWA with an aim to develop culturally responsive graduates.

This study aims to evaluate the impact of an Aboriginal and Torres Strait Islander health educational webinar on Australian medical students’ Indigenous health knowledge and cultural humility. Student-led evaluation and feedback should form part of continual improvement in respect to Indigenous health medical education within Australian medical schools. Research evaluating the short- and long-term impact of cultural humility webinars would be an invaluable part of improving education and teaching to change attitudes and perceptions among future healthcare workers.

## Methods

We used quantitative and qualitative research methods to evaluate student learning outcomes from an online educational IH cultural humility webinar. The webinar curriculum was developed by the Australian Medical Student Association (AMSA) and the General Practice Student Network (GPSN) in collaboration with members of the Indigenous community and in reference to the Aboriginal and Torres Strait Islander Health Curriculum Framework [5]. The webinar was delivered by members of the Indigenous community using a common online video meeting platform over a duration of two hours.

Our quantitative analysis involved survey questionnaires conducted immediately pre- and post-webinar. The pre-webinar survey was distributed at the beginning of the webinar. Post-webinar surveys were sent out immediately after and three-month after the webinar to assess knowledge retention. Survey questions were developed to reflect the pre-determined learning objectives (LO) of the webinar (see Table 1).

**Table 1:** Learning Outcomes Explored in the Survey Questions

Learning Outcomes	Components
1. Defining the gap and addressing the disparity	Origins of health inequality The social determinants and impacts Lifestyle and resource availability and Aboriginal and Torres Strait Islander health
2. Exploring the links between health and education	Self-reflection and why it is important Beliefs, assumptions and perceptions and the impact on practice Incorporating cultural humility and responsive into healthcare settings
3. Preparing for placement within community	What to know before placement The role and impact of Aboriginal medical services How you can be part of the solution The importance of empowerment and a strength-based approach

Our qualitative analysis involved a semi-structured interview conducted approximately one month after the webinar over an online video meeting platform. Participants were selected from volunteers that expressed interest in participating in the semi-structured interview. The duration of the interview was approximately one hour with questions developed to prompt participants to reflect on the positive and negative aspects of the webinar.

### Ethical consideration

This study was approved by the Bond University Human Research Ethics Committee (SR00253). During recruitment, participants were provided with a plain language statement and consent form detailing the purpose of the study, ethical consideration, study involvement, study withdrawal and data handling considerations.

### Participant recruitment

Medical students currently enrolled in an Australian medical degree from any approved Australian medical school were permitted to participate in the study. We excluded medical students studying overseas and those under the age of 18 years. The webinar and registration link were advertised on social media pages hosted by GPSN and AMSA. The registration link for the webinar allowed students to voluntarily participate in the study and the webinar.

### Data collection

Quantitative data included participant demographic and survey question responses collected through online Google Form submissions. The survey involved twenty multiple choice questions. Each question included multiple correct and incorrect responses and participant scores were based on the number of correct responses selected. Participant scores were calculated as a percentage based on the number of correct responses selected. To maintain anonymity, investigators were blinded and an independent third party generated an identification number for each participant to reference in their survey response submissions for retrospective comparison between the pre- and post-webinar responses. The matching of identification numbers to pre- and post-webinar survey responses were conducted by an independent third party to produce a finalised de-identified dataset for analysis by the study authors. Qualitative data was collected as a video recording of the focus group discussion. The recording was independently reviewed by the researchers with recurring themes identified and documented.

### Data analysis

We analysed data using SPSS. Pre- and post-webinar scores were analysed by frequency, mean and standard deviation. A paired t-test was used to compare continuous variables and chi-squared test to compare categorical variables. The semi-structured interview allowed participants to reflect on positive and negative aspects of their experience during the webinar. Common themes were identified as part of this discussion and reported on as part of qualitative data to support other components of the research.

## Results

### Quantitative

#### Participant demographics

Seventy-five Australian based medical students registered and consented to participate in the study. Of these, thirty-three participants chose to participate. Due to incomplete responses on the pre- and immediate post-webinar surveys, seven participants were excluded from the study. Therefore, twenty-six participants were included in the final analysis for the immediate results. All participants were lost to follow-up for the three-month survey.

Participant age was stratified in three age groups with most participants aged between 18 and 24 years of age (n = 18, 69.2%). Six participants in the age group of 25–30 (23.1%) and 2 participants in the age group of 31–36 (7.7%). There were 20 female participants (76.9%) and 6 male participants (23.1%). There were 17 clinical participants (65.4%) and 9 pre-clinical participants (34.6%). All the participants identified as Non-Aboriginal or Torres Strait Islander (see Table 2).

**Table 2:** Demographic Characteristics

Demographics	Participants in Webinar Survey (n = 26)
Age	
18–24	18
25–30	6
31–36	2
Gender	
Female	20
Male	6
Clinical Status	
Pre-Clinical	9
Clinical	17
Identified as Aboriginal and Torres Strait Islander	
Yes	0
No	26

Chi-square analyses were conducted to determine the association between the individual demographic characteristic and survey scores. No significant associations were found between age group and pre-intervention score (*Χ*^2^(2) > = 16.972, *p* = 0.525); gender and pre-intervention score (*Χ*^2^(2) > = 10.508, *p* = 0.311), or clinical status and pre-intervention score (*Χ*^2^(2) > = 8.879, *p* = 0.449). No significant associations were found between age group and immediate post-intervention score (*Χ*^2^(2) > = 11.074, *p* = 0.805); gender and immediate post-intervention score (*Χ*^2^(2) > = 4.406, *p* = 0.819), and clinical status and immediate post-intervention score (*Χ*^2^(2) > = 9.800, *p* = 0.279).

#### Webinar efficacy

**Fig. 1:**
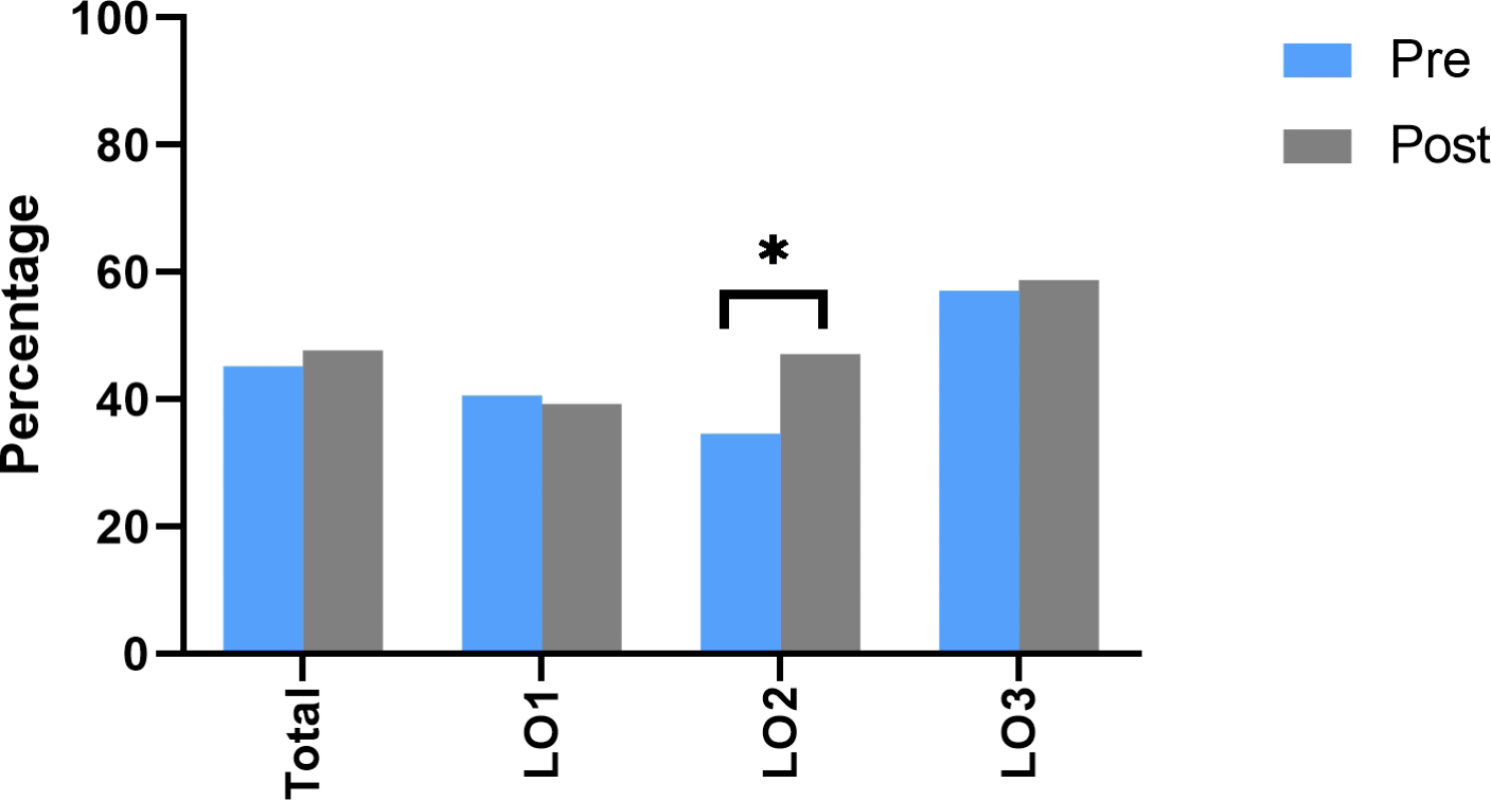
Change in pre- and immediate post-intervention scores for total and stratified by learning outcome. There was a significant difference between pre- and immediate post- survey result when comparing questions related to learning outcome two

There was not a statistically significant increase (*p* = 0.196) in the total participant’s score from pre-intervention (*M* = 0.45, SD = 0.11) to the immediate post-intervention score (*M* = 0.48, SD = 0.13). However, stratification based on learning outcomes demonstrated a significant increase (*p =* 0.007) between pre-intervention (*M* = 0.35, SD = 0.26) and post-webinar knowledge for the second learning outcome (*M* = 047, SD = 0.25) (see Fig. 1).

### Qualitative

Three volunteers from the webinar attendance list participated in a focus group discussion facilitated by one of the research authors. Focus group members reflected on the inconsistency of IH teaching between Australian medical schools. They noted the importance of extracurricular events to supplement their learning in respect to cultural humility.

My university doesn’t do a lot, so I seek out webinars and seminars to get more insight and feel more prepared.

The webinar was very well organized and the speakers had some powerful messages.

There were a lot of topics to cover and there was a clear outline for the webinar.

The focus group members unanimously agreed that the speakers and their method of presentation was a critical part of the webinar’s success. The conversational method of yarning was preferred over more a didactic delivery of learning materials.

I felt that having several engaging speakers was an important element as it provided alternative perspectives on the topic.

The key to a webinar is the speakers because it really depends on how the speakers interact and engage with the audience.

The group members noted that access to webinars from anywhere in Australia was a primary benefit for the online format.

I can access a lot more webinars than I would.

It’s really great to be able to attend webinars from all over Australia.

One group member noted that the online format perhaps limits the level of interaction that you get from an in-person event.

## Discussion

Our quantitative results showed an insignificant increase between mean pre- and immediate post-webinar overall knowledge scores (*p* = 0.196, *M* = 0.45, SD = 0.11). Follow-up analysis showed a significant increase between (*p* = 0.007) mean pre and immediate post intervention score for the second learning outcome. This learning outcome explored the links between health and education and required participants to reflect on beliefs, assumptions, and perceptions to respond correctly to the survey questions. Our qualitative results suggest that this may be attributed to the presenter delivery style. There was consensus that the conversational method of yarning was preferred over a more didactic and structured delivery of the curriculum because it promoted greater reflection on personal bias and assumptions.

It is possible that medical student knowledge for the first and third learning outcomes may have been high prior to the webinar, as curriculum related to Indigenous health inequality has been prioritised by the Australian government [12]. Additionally, the webinar questions for the first and third learning outcomes may have been an inadequate evaluation tool in this setting. Some suggest that the multiple-choice quiz requires consideration of the existing strengths and weaknesses of the student cohort to be a valid evaluation tool [13]. Our study did not consider the existing level of knowledge among the participants. We suggest this as an important factor during the selection and design of tools for evaluation of future Indigenous health education initiatives.

Each speaker was asked to focus on a particular learning outcome and asked to present their information at a pre-determined time during the webinar. Giles et al. [14] studied medical student engagement levels during lectures and found that information presented fifteen to thirty minutes into a session is best recalled by medical students. Material related to learning outcome two was delivered thirty minutes after the commencement of the webinar. The significant improvement in knowledge for this learning outcome may have been due to the optimal time of delivery, independent of speaker presentation styles.

Our study provides a unique example of student-led research investigating outcomes related to Indigenous health education. Warren et al. [15] used pre- and post-education questionnaires to evaluate medical student learning outcomes from clinical placements in remote Indigenous communities. However, financial and contextual barriers prevent many students from participating in remote clinical placements during their health studies [16]. Our study suggests that online webinars are a classroom based educational tool that can improve student understanding of questions related to Indigenous culture, kinship, and land. For example, *“What are the core aspects of Aboriginal and Torres Strait Islander kinship?”* and *“ In what ways has Aboriginal care been impacted by the beliefs, assumptions and perceptions held by health professionals?”*

### Limitations

Recruitment of participants for the qualitative study arm was done on a voluntary basis and therefore the qualitative cohort was not necessarily representative of all webinar attendees. Participants with greater interest in the webinar content were more likely to attend and those who had enjoyed a positive webinar experience may have been more encouraged to further lend their time to the study.

IH education varies widely between medical schools and year of study and participants may have come to the webinar with widely varied knowledge and understanding. Future studies should consider this cohort variability and recruit participants based on their current level of understanding and learning expectations.

Sample size was a significant limitation for our study. Loss to follow-up of all participants at three months post the intervention prevented an analysis of knowledge retention over a longer period. Recruitment of a larger cohort would allow greater scope for data collection and analysis to identify specific elements of the intervention that had the greatest impact on participant knowledge and understanding.

The survey questions were developed to directly reflect the content of the webinar curriculum and allow accurate analysis of the intervention. This could have been made clearer to participants at the beginning of the webinar as the presentations allowed for individual participant interpretation.

### Strengths

The inexpensive cost and simple methodology of our study present as key strengths. The project was led entirely by Australian medical students with diverse experience and a common motivation to improve IH education. The study used freely available online resources for survey, webinar delivery and data analyse. The studies mixed-methods approach combined quantitative analysis with a qualitative interview providing a nuanced picture of webinar impact.

The curriculum of our webinar was based on the Aboriginal and Torres Strait Islander Health Curriculum Framework [5] allowing for our findings to be more relevant and tailored to the national IH agenda. Furthermore, we collaborated extensively with several members of the Indigenous community, including medical students and doctors, to ensure the highest level of cultural sensitivity throughout our project.

### Recommendations

Our results suggest that online webinars may be an effective platform for the delivery of culturally sensitive discussions. Our qualitative analysis suggested that student engagement and interaction was significantly greater with the more flexible and personable style of conversational yarning. Clinical yarning has been described as an effective method of developing a therapeutic relationship with Indigenous patients [17]. Our results suggest that yarning may be an effective method for teaching medical students about abstract elements of cultural humility such as self-reflection and bias. Future empirical studies are necessary to replicate these findings across a larger sample size.

A significant consideration in the delivery of IH education is the participation and representation of Indigenous people. The lived experience of the Indigenous facilitators was discussed by the authors as a critically important element of the webinar and method of delivery. Our pilot study has demonstrated how the encouragement and facilitation of reflective learning can improve the knowledge and understanding of Australian medical students about Indigenous kinship, culture, and land. Insights from this study support a mandate for increased participation of Indigenous people in the development and delivery of IH teaching.

## Conclusions

This study used quantitative and qualitative research methods to demonstrate the efficacy of an Aboriginal and Torres Strait Islander cultural humility webinar on the knowledge and understanding of a small cohort of Australian medical students. We found a significant improvement in medical student knowledge and understanding about Aboriginal and/or Torres Strait Islander kinship, culture and land after attending an online IH webinar. The importance of confident and engaging webinar facilitators in the delivery of IH webinars should not be understated and was suggested by our study as an important element. Students’ preference for a conversational method of teaching over traditional didactic methods of curricula delivery is a critically important consideration when designing future IH education webinars.

This pilot study warrants further empirical research studying the impact of cultural humility webinars on Australian medical students. A comprehensive understanding of IH cultural humility among Australian medical students is vital to improve cultural humility within the Australian health system. Importantly, students must be self-aware about the impact of their beliefs and assumptions on interactions with Aboriginal and/or Torres Strait Islander peoples. Further research in this space is needed to develop effective IH curriculum and ensure all Australian medical students graduate as culturally reflective practitioners. We hope our research is an encouraging example for future student-led initiatives.

### Electronic supplementary material

Below is the link to the electronic supplementary material.

Supplementary Material 1

## Data Availability

The datasets used and analysed during the current study are available from the corresponding author on reasonable request.

## List of Abbreviations

AMSA: Australian Medical Students’ Association
GPSN: General Practitioners Student Network
MDANZ: Medical Deans of Australia and New Zealand
UWA: University of Western Australia
IH: Indigenous Health
SPSS: Statistical Package for Social Sciences
