# Supplementary material for: A pilot study to assess the impact of Aboriginal and Torres Strait Islander cultural humility webinars on Australian medical school students
Source: BMC Med Educ. 2023 Sep 3;23:626. doi: 10.1186/s12909-023-04612-7 (PMC10476379; doi:10.1186/s12909-023-04612-7)
Supplement: Supplementary file 1 — Supplementary Material 1 [file 12909_2023_4612_MOESM1_ESM.docx]

**APPENDICES**

**Appendix 1.** Questionnaire

*Questionnaire*

The following questions have been designed according to resources from the Australian Institute of Health and Welfare, peer-reviewed medical journals and Australians Together. They also reflect the themes and learning outcomes of our webinar. Each question will be multi-choice, with the option for respondents to tick more than one answer. The correct answers have been highlighted with red text for reference.

1. We understand that social determinants of health and behavioural risk factors both contribute to the health gap, but **how is the health gap defined**?
   1. More expensive consults for certain target groups
   2. Decreased life expectancy
   3. Higher incidences of chronic disease in the Indigenous population
   4. Inadequate access to healthy foods
   5. Genetics
   6. Unequal access to care lies propagated by the government
2. Which of the following clinical questions demonstrates a strength-based approach?
   1. “What does this condition hinder in your day-to-day life?”
   2. “Which parts of your condition are you managing well?”
   3. “What’s the most important aspect of treatment that you’re concerned about?”
   4. “What do you find the worst about your condition?”
3. Of the following which best describes the **reasons behind the disparity** in health outcomes for Indigenous peoples?
   1. Continued discrimination as well as atrocities of the past have resulted in long-term, trans-generational negative physical and psychological health outcomes
   2. Increased health risk behaviour for Indigenous peoples is a conscious choice that is within their control
   3. The culture of the Australian healthcare system has no role in the health disparity experienced by Indigenous peoples
   4. Disadvantages in the social determinants of health and many other facets of life have continued to affect the health outcomes for members of the Indigenous population
   5. Culturally inadequate and poor healthcare access has also contributed to the observed health disparity
4. What are the **top 4 behavioural risk factors** that contribute to the health gap between Indigenous Australians and non-Indigenous Australians?
   1. Smoking
   2. Alcohol
   3. Illicit substances
   4. Physical inactivity
   5. Diet
   6. Obesity
   7. Unemployment
5. Do **social determinants or behavioural risk factors** play a bigger role in accounting for the health gap between Indigenous and non-Indigenous peoples?
6. Social determinants
7. Behavioural risk factors
8. What are the three factors that directly influence the **lack of access** to healthcare for many Indigenous Australians?
   1. Life expectancy
   2. Affordability
   3. Poor health
   4. Laziness
   5. Cultural safety
   6. Genetics
   7. Distrust
   8. Limited support networks
9. Indigenous peoples have **limited access** to which of the following resources?
10. Specialised health care
11. Ambulatory services
12. Culturally appropriate health care
13. Proximal health care
14. Access to primary health care
15. Which of the following is true for **lifestyle factors** experienced by Indigenous peoples?
    1. Greater proportion of rural living compared to the non-Indigenous population
    2. Similar rates of obesity compared to non-Indigenous peoples
    3. Higher rates of tobacco smoking
    4. Poorer rates of nutrition due to socio-economic, social, and environmental factors
    5. Maintain strong social connections with kin
16. How do the Aboriginal and Torres Strait Islanders differ in their **culture** from Western society?
    1. Emphasis on animism (everything is interconnected, people, plants and animals, landforms and celestial bodies are part of a larger reality)
    2. Belief that the Dreamtime is the origin of the universe
    3. Emphasis on individual development over the community
    4. There is a mutual respect and understanding of roles within a community
    5. Emphasis on totem - natural object, plant or animal that is inherited by members of a clan or family as their spiritual emblem. Totems define peoples' roles and responsibilities, and their relationships with each other and creation.
    6. Role of connectedness and responsibilities for other people (their kin), for country (including watercourses, landforms, the species and the universe), and for their ongoing relationship with the ancestor spirits themselves.
    7. Emphasis on the present aspect of time - on the here and now.
17. Which of the following are core aspects of Aboriginal and Torres Strait Islander **kinship**?
18. A person’s relationship to their whole family—not just to their parents and siblings
19. A person’s relationship to their community—not just their family
20. A person’s relationship to the land and the spirit beings which determine lore and meaning.
21. A person’s relationship to their vocation.
22. In what ways has Aboriginal care been impacted by the **beliefs, assumptions and perceptions** held by health professionals?
23. Not creating a safe space for questions to be asked
24. Not delivering information in a way that can be understood, such as the inclusion of medical jargon or not utilising an interpreter
25. Delivering culturally irrelevant health education materials
26. Deliberately prolonging wait times in healthcare settings
27. Not allowing time for patients to process information
28. Which of the following are appropriate **questions** to consider in assessing an individual’s cultural competency and cultural preparedness?
29. “When I see a patient from a culture unfamiliar to me, I seek information about his/her culture”
30. “I should provide all patients with the exact same standardised treatment, including the services that I refer to, education materials that I provide, and the words used in my explanation”
31. “How prepared do you feel to care for patients with a distrust of the healthcare system?”
32. “I adapt my care to patient’s preferences”
33. “What influences my core values and beliefs and how does this differ from my patients?”
34. Which of the following would **best improve Indigenous health outcomes**?
35. Implementing more Indigenous-specific health care services which address cultural competence
36. Telling these communities to change their lifestyle
37. Acknowledging the multidimensional nature of health care access and considering a variety of factors when implementing changes
38. Addressing physical and geographical barriers through strategies such as implementing local health services and facilitating transport to services
39. Providing a more structured health service system that has a large focus on the biomedical aspect of health
40. How can this gap be addressed?
41. Addressing Year 12 completion rates
42. Working with Aboriginal healthcare workers and empowering them to improve health and wellbeing
43. Campaigning for Indigenous peoples to stop smoking / drinking alcohol
44. Providing more employment opportunities
45. Consulting Aboriginal leaders and representatives to provide more funding and mentoring opportunities
46. Which of the following **actions demonstrate cultural awareness** for interactions with First Nations Peoples in a clinical setting?
47. Acknowledging the importance of family and community
48. Striving for community-centred attitudes and treatment options
49. Ensuring clear breakdowns of service pricing and delivery
50. Having an awareness of whose country you are working on
51. Punctuality and professionalism in visits and appointments
52. What are some of the **core values** of Aboriginal and Torres Strait Islander Peoples?
53. Family & kinship
54. Community
55. Material wealth
56. Connection to land & spirituality
57. Nihilism
58. Which of the following actions embodies a core principle of respectful and **holistic health** care for Aboriginal and Torres Strait Islander peoples?
59. Actively involving and engaging patients in discussions regarding their healthcare
60. Consulting with and obtaining consent for all procedures with clear communication with all patients
61. Treating patients the same regardless of race, gender or circumstance
62. Remaining mindful of upstream determinants which may hinder primary care and treatment
63. Which of the following define the services that **Aboriginal health workers** provide?
64. Community liaison and engagement
65. Advocacy
66. Health promotion and education
67. Culturally safe services
68. Cultural education and brokerage
69. Community development
70. Disease prevention
71. Which of the following are major benefits of a **strength-based approach** to medicine?
72. Mutual relationship between patient and practitioner
73. Development and maintenance of a sense of wellbeing
74. Nurturing of physical and mental strength under a strict regime
75. Allowing patients to ignore their shortcomings
76. Why is a **strength-based approach** particularly relevant for Indigenous communities?
77. The strength of Indigenous communities as a whole towards incredible adversity should be recognised and celebrated
78. Aboriginal and Torres Strait Islander peoples value strength above all else
79. It aligns with the principles of empowerment, healing and self-determination
80. Indigenous communities do not respect other forms of service
